# Improving nutrition for migrant children in Europe through policy: A scoping review^☆^

**DOI:** 10.1016/j.jmh.2024.100290

**Published:** 2025-01-06

**Authors:** Rebecca Lawes, Professor Debbi Marais, Professor Mariza Louw, Ms Beatrice Bennett

**Affiliations:** University of Warwick, UK

**Keywords:** Refugee, Asylum seeker, Child, Migrant, Nutrition, Intervention

## Abstract

Nutrition research concerning migrant populations is abundant, with a surplus of prevalence data related to diverse aspects of malnutrition. However, there is a lack of studies on European country interventions. This gap is more pronounced when examining interventions specific to children. The challenges are clear, but the policy impetus for solving them is not. Examining European interventions on nutrition in refugee and asylum-seeking (RAS) children and families allows for an assessment of the unmet needs in the United Kingdom regarding migration, nutrition, child support, and food security.

This scoping review followed the guidelines in the PRISMA Extension for Scoping Reviews, and 20 documents which covered a mixture of policy interventions were eligible. Meaningful comparisons can be made by assessing the interventions made in migrant nutrition from various European countries with varying political approaches to immigration. This creates a fuller picture of the policy landscape for paediatric migrant nutrition, and in due course, policy decisions can be taken.

This scoping review found a lack of diversity in RAS nutrition policies across Europe and a lack of transparency in those in place, likely rooted in the challenging political landscape of increasing RAS populations in Europe. Improving nutrition interventions requires separating food and health from politics. No interventions focusing on the double burden of malnutrition were found. However, policies addressing the double burden in RAS children should be considered as they may be more politically palatable and financially beneficial to implement.

## Highlights

• Examining European interventions on nutrition in refugee and asylum-seeking (RAS) children and families allows for an assessment of the unmet needs in the United Kingdom regarding migration, nutrition, child support, and food security.
• There is a lack of diversity in RAS nutrition policies across Europe and transparency in those in place.
• Issues are rooted in the challenging political landscape of increasing RAS populations in Europe. Improving nutrition interventions requires separating food and health from politics.
• No interventions focusing on the double burden of malnutrition were found. However, policies addressing the double burden in RAS children should be considered as they may be more politically palatable and financially beneficial to implement.

## Definitions

In this study, **refugee and asylum-seeking (RAS) children** will be used as a term to refer to children and adolescents younger than 18 who were moving across borders and have settled in a country different from their place of birth after experiencing unfavourable conditions, including exposure to war and other forms of violence, socioeconomic deprivation and limited access to health care and education (Health, 2018). This covers both unaccompanied children in the care of the state of the host country and those living with their families. This definition includes children from families with recognised refugee status and people who are currently undocumented but are residing in a host country (UNICEF 2023).

## Background

Nutritional concerns, including malnutrition, overnutrition and lack of micronutrients, are a problem for refugees and people seeking asylum (RAS) living in Europe (Ankomah et al., 2022). The importance of adequate nutrition for all children and young people is well documented. It can equate to healthier adulthood, lower burden of non-communicable disease (Di Daniele, 2019), and better educational attainment and productivity (Nyaradi et al., 2013), making the public health impetus of the area a priority.

The double burden of malnutrition is the existence of overnutrition, including overweight and obesity, alongside undernutrition, which can include stunting and wasting at all levels of the population—and in individuals over a life course (The Lancet 2019). Evidence suggests an emerging triple burden of malnutrition where a lack of micronutrients in an individual's diet is also captured (Kumar et al., 2021).

Within this global nutrition backdrop, the health and well-being of migrants are increasingly becoming a focus point in Europe. There are more RAS than ever due to several geopolitical crises (Nisbet et al., 2022), including war in Ukraine, ongoing political unrest across parts of the Middle East, Israel and Gaza and conflict in Syria, and increasing numbers of natural disasters, including earthquakes and flooding relating to climate change (Concern Worldwide 2023; United Nations High Commissioner for Refugees 2023). Eighty percent of the world's displaced people are from locations suffering from acute malnutrition or food insecurity (United Nations High Commission for Refugees 2019), and >90 % of the world's refugees are settled in high-income countries, including Europe (United Nations High Commission for Refugees 2019). Between 2012 and 2022, two million children filed for asylum status for the first time in Europe (Eurostat 2023). This study, therefore, sits at the intersection between one of the most significant public health topics globally – nutrition – and a subset of the most vulnerable people in Europe – RAS children. This study maps existing policies across Europe in place to address food insecurity and malnutrition in RAS children in Europe to make recommendations for additional and strengthened policies.

The importance of a nutritious diet for public health is widely understood and becomes even more critical for migrant populations (Wood et al., 2021). Studies exploring food insecurity habits and culture amongst refugees in European countries are plentiful (Terragni et al., 2014), but interrogation of food policy initiatives impacting refugees is less so.

Previous scoping reviews have investigated refugee nutrition approaches in high-income countries, but these focus predominantly on food security (Wood et al., 2021) and the cultural sensitivities contributing to it (Gingell et al., 2022). The most prominent study in the area focused on intervention is from Nisbet et al. (2022) (Nisbet et al., 2022), a global study of food security interventions for refugee communities. However, there is a notable gap in their research concerning European countries and children and families.

Nutrition is of great concern in RAS children due to the immediate risk from malnutrition and the potential for a pre-existing lack of essential micronutrients due to long travel times or fleeing challenging environments (Krishnamani, 2016). Migrant children face a higher risk of obesity, especially in Western societies, due to acculturation and lifestyle changes (Gualdi-Russo et al., 2014), and migrants often adopt Westernized diets. These are associated with increased risk and prevalence of obesity with high sugar, fat and salt content, needing to opt for low-cost, high-calorie food and needing to abandon their traditional food habits (Gilbert and Khokhar, 2008). RAS children are less likely to be able to learn cooking and food practices from their parents due to being on the move, and cultural food identities can be lost (Berggreen-Clausen et al., 2022). A change in diet and food practices is also associated with an increased risk of non-communicable diseases in RAS migrating to high-income countries (Agyemang and Van den Born, 2019).

Assessment of policy and interventions for nutrition in RAS children and young people is the primary gap in research currently, with explorations of nutrition interventions in this specific group found in the US (Lopriore et al., 2004) and Australia (Fabio, 2014) but lacking in Europe. A similar review into child migrant health, which looked at health issues and non-communicable diseases in 2019, had a similar observation about the geography of intervention-focused studies (Kadir et al., 2019).

Concern about the lack of research into interventions to improve nutrition and micronutrient intake urgently upon arrival should not overshadow a broader need to focus on nutrition and the more comprehensive health needs of RAS over the life course. Nutrition is inextricably linked with well-being and the determinants of migration upon health, such as being less likely to be vaccinated, being born outside of a medical setting, physical health problems occurring through travel and psychological impacts (for example, trauma is observable in almost all RAS children) (Fazel and Stein, 2002). This is relevant because whilst interventions relate to the practicalities of eating, drinking and accessing nutrients, the reality of the limitations in refugee nutrition can often fall beyond practical access: having trauma around food, cultural insecurities, language barriers, social marginalisation and issues with self-esteem which manifest through food (ISSOP Migration Working Group 2018).

The objectives of this study were to:

A) identify policies and interventions that are in place for RAS child nutrition in different European countries
B) evaluate whether the policies are implemented
C) assess the impact of the interventions on the double burden of malnutrition
D) develop recommendations to improve nutrition in the chosen population

This study aims to fill a current gap in European research for both population area and age of the subject. The study's interest in interventions from the perspective of policy comparison has the potential to inform tangible system change in an essential area of public health in the UK and across Europe. A scoping review following the guidelines in the PRISMA Extension for Scoping Reviews (Tricco et al., 2018) and structural inspiration from the scoping review guidelines proposed by Arksey and O'Malley (Arksey and O'Malley, 2005) was conducted on nutritional interventions which target children and their families who are refugees or seeking asylum in Europe.

## Methods

### Search strategy

The following search strategy, including key search terms and Boolean operators, was used for database searches, which was developed alongside an academic librarian to ensure that appropriate documents would be captured by the search: (((((refugee) OR (asylum seek*)) OR (migrant*)) AND (malnutrition OR nutrition OR double burden malnutrition OR obesity)) AND (child OR adolescent OR teenage OR family)) AND (policy OR strategy OR intervention) AND Europe

Six academic databases were searched: PubMed, CINAHL, Scopus, Ovid, Web of Science, and Applied Social Science Databases. The grey literature was also searched because the review concerns interventions and national policies, which tend to be unpublished and not captured in academic database searches (Adams et al., 2016).

As part of the grey literature review, data was sourced from the following places:

Google Scholar – Capturing of first 3 pages

• OpenAIRE for full search term (see above)
• World Food Programme website search for “child refugee nutrition policy Europe”
• United Nations website search for “child refugee nutrition policy Europe”
• United Nations High Commission for Refugees (UNHCR) website search for “child refugee nutrition policy Europe”
• Selected country government website searches “child refugee nutrition policy.”
• For webpages, 3x search pages were reviewed in detail, with relevant articles of interest given a full-text review.

### Screening

This study looked at European countries’ approaches to tackling malnutrition (low-weight-for-height), overnutrition (high-weight-for-height) and lack of micronutrients in RAS children of all ages settled in European host countries (Table 1). It included interventions targeted at or impacting the nutrition of RAS children and their families living in state-provided accommodation and interventions tackling wider nutritional challenges for those settled in communities. RAS families are the focus, but relevant interventions for people of other migrant status were also included where the intervention was relevant. This study included grey and traditional literature, including but not limited to government policy documentation, independent policy reports, case studies, charity and non-government organisation (NGO) press releases and website copy, as well as relevant studies, including mixed method reviews and qualitative interview data.

**Table 1:** Eligibility criteria for scoping review of European country interventions for nutrition improvement in refugee and asylum-seeking children and their families residing in Europe.

Inclusion	Exclusion
• Studies about children and adolescents and their families of all ages (1–18)	• Data on nutrition which doesn't impact refugee or migrant children
• Studies covering RAS from any country of origin • Studies covering eligible age group refugees or people of registered asylum status	• Prevalence data relating to malnutrition in which policies or interventions are not mentioned • Interviews and perspectives of refugees and migrants where interventions and recommendations are not referred to or prevalence is the focus
• English language documents only	• Documents which were published before 2000
• Grey literature fulfilling the search criteria which could include: ○ Government policy documentation ○ Independent policy reports ○ Charity and NGO press releases and website copy ○ mixed method reviews qualitative interview data	

Rayyan was used to retrieve documents from the database search, and duplicates were resolved before screening was initiated. The systematic screening process of academic database searched items was undertaken in two phases, namely title and abstracts and then full-text review, independently by two researchers (RL and BB), and a third reviewer (DM) reviewed any disagreements at each phase. The additional grey literature items were reviewed by one researcher (RL) using the same eligibility criteria. A small amount of handsearching was then undertaken, where documents were referred to in other documents and retrieved.

## Data extraction

The following information was extracted from each document: first author's surname, name of document, date of document, citation, and type of document (specifically relating to whether it is a national or international policy, guidance, recommendation or criticism).

## Data analysis

Each document was assessed in line with the four objectives noted previously, with policies and interventions and specific recommendations within each document synthesised according to the objective and narrative analysis performed.

## Results

**Fig. 1:**
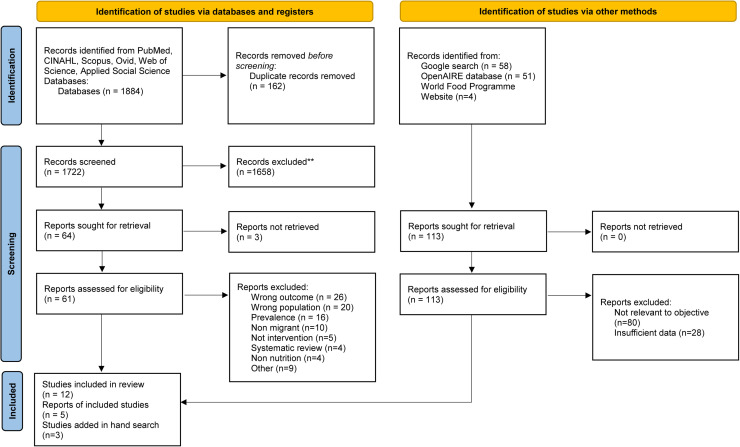
PRISMA flow diagram of the screening of relevant articles to explore European policies and interventions impacting the nutritional status of refugee children and their families settled in Europe.

The six academic databases yielded 1884 records, and the grey literature searches yielded 113 (Fig. 1). After duplicates were removed, there were 1722 documents from academic electronic databases. An initial screening using the abovementioned criteria eliminated 1658 documents, leaving 64 for full-text review. The 113 records retrieved through grey searches were screened to five relevant documents, with an additional three added through a hand search.

At the full-text review stage, for the database search, the predominant reasons for exclusion were wrong outcome (*n* = 26), wrong population (*n* = 20), and studies that showed prevalence data rather than interventions (*n* = 16). For the grey search, the criteria for exclusion were limited to lack of relevance to objectives and insufficient data only, of which 80 and 28 documents, respectively, were excluded at the full-text review stage.

Twenty documents were eligible and included in the data extraction and analysis for this study.

## Report characteristics

A quarter of the 20 documents included were from the UK, 20 % were from Greece, 10 % were from Germany, and 5 % were from both Switzerland and Italy, with the remaining 35 % being global or European-wide documents (Table 2).

**Table 2:** Extracted documents organised by lead author or organisation name, title of document, country or region date of document, categorisation of document type.

**Number**	**Lead author**	**Title**	**Country or region**	**Date of document**	**Categorisation**
1	Kahn Shazia, et al	Nutritional habits of asylum seekers living in communal accommodation in Stuttgart, Germany	Germany	2018	National policy or guidance
2	UNHCR	Infant and Young Child Feeding in Emergencies - Operational Guidance for Emergency Relief Staff and Programme Managers	Global	2017	International policy
3	Office for Health Improvement and Disparities	Children's health: migrant health guide and healthy start programme UK	UK	2014	National policy or guidance
4	Taylor, Diane	Children in England's asylum hotels suffering from malnutrition	UK	2023	Policy analysis or criticism
5	Federal Ministry for Economic Cooperation and Development	Agents of Change: Children's and Youth Rights in German Development Cooperation	Germany	2017	International policy developed by a national government
6	Intersos GR	Being Hungry in Europe: An analysis of the food insecurity experienced	Greece	2023	Policy analysis or criticism
7	UNHCR	Food and Nutrition Operational Considerations for Refugee and Migrant settings in Greece	Greece	2016	National policy or guidance
8	Smith, Helen	Greek government blamed for hunger crisis in refugee camps	Greece	2022	Policy analysis or criticism
9	ISSOP Migration Working Group	ISSOP position statement on migrant child health	Europe wide	2018	International policy
10	Harkensee, Christian, and Andrew, Rachel.	Health needs of accompanied refugee and asylum-seeking children in a UK specialist clinic	UK	2021	National policy or guidance
11	Schrier, L., Wyder, C., del Torso, S. et al	Research and the promotion of child health: a position paper of the European Society for Pediatric Gastroenterology, Hepatology, and Nutrition.	Europe wide	2014	International or Europe wide policy
12	Schrier, Lenneke et al	Medical care for migrant children in Europe: a practical recommendation for first and follow-up appointments	Europe wide	2019	National policy or guidance
13	Prudhon, C., Maclaine, A., Hall, A. et al	Research priorities for improving infant and young child feeding in humanitarian emergencies	Global	2016	Policy analysis or criticism
14	Park, Minhye et al	Child and adolescent health in Europe: Towards meeting the 2030 agenda	Global	2023	International or Europe wide policy
15	Office for Health Improvement and Disparities	UK Government guidance - Children's health: migrant health guide	UK	2014	National policy or guidance
16	UNICEF	Deep dive - Child Gaurantee - Analysis of politics, programmes, budgets addressing child poverty and social exclusion in Italy (nutrition chapter)	Italy	2022	National policy or guidance
17	European Commission	Handbook for health professionals: Health assessment of refugees and migrants in the EU/EEA	Europe	2015	International or Europe wide policy
18	Bernhard, Sara et al.	Guidance for testing and preventing infections and updating immunisations in asymptomatic refugee children and adolescents in Switzerland	Switzerland	2016	National policy or guidance
19	RCPCH	RCPCH: Refugee and asylum seeking children and young people - guidance for paediatricians	UK	2018 (updated 2023)	National policy or guidance
20	UNHCR	Multi-Purpose Cash and Sectoral Outcomes - Case Study Greece	Greece	2018	Policy analysis or criticism

Almost half (45 %) of the 20 documents were national policies or guidelines applicable at a country level, 30 % were international or Europe-wide policies or guidelines, and the remaining 25 % were categorised as policy analysis, criticism or recommendation. Of the criticism and analysis articles, one was a news report (Taylor, 2023), one was a report written by an NGO (Intersos, 2023), and the other was a UNHCR case study into an intervention on cash schemes for refugees and migrants (UNHCR 2018).

Almost a third (30 %) of the documents included screening interventions such as health professional guidance for assessing the health (and nutritional status) of migrants upon entry to an arrival country. A quarter (25 %) of documents focused on increasing breastfeeding rates as a priority intervention. Cash assistance as a food security and nutrition support intervention was noted in 15 % of documents.

Only one document (UNHCR 2016) provided data for all objectives (Table 3). Most, but not all documents provided some data toward objective one “**identify policies and interventions that are in place for refugee child nutrition in different European countries**” and few documents provided any data for the third objective “**evaluate the impact of the interventions on the double burden of malnutrition”.**

**Table 3:** Documents sorted into data extraction correlating to objectives A,B,C,D.

Table 3 provides a visual aid indicating which papers’ data corresponds to which objective. It shows that objective three, evaluate the impact of interventions on addressing the double burden of malnutrition is seldom addressed.

### Policies and interventions that are in place for RAS child nutrition in different European countries

#### Nutritional status screening

Policies in place to improve RAS child nutrition include screening by a healthcare professional on arrival to host countries and tailored health advice depending on screening outcomes. This was noted specifically for the UK (Office for Health Improvement and Disparity 2014) and in EU-wide articles due to migrant children being treated the same as non-migrant children by EU law (UN Convention for the Rights of the Child) (ISSOP Migration Working Group 2018). Checking for growth and development and testing for iron deficiency, nutritional rickets and vitamin D deficiency is recommended across several European countries (Schrier et al., 2019) and is noted specifically in the UK guidance (Office for Health Improvement and Disparity 2014). In Switzerland, screening takes place, but nutrition is only observed indirectly through checking for parasites and testing stool samples; rather, vaccine and infectious disease status are prioritised at the screening stage (Bernharda et al., 2016).

Nutrition screening is listed as part of “observations” in EU documentation except for UK guidance, which specifically recommends assessment for malnutrition (RCPCH 2018). However, this is guidance for paediatricians and not medical law.

#### Cash assistance

Cash assistance is a policy in some areas of Greece to support RAS families (UNHCR 2018). Usually implemented with the support of charities and NGOs supporting ‘on the ground efforts’, cash programmes can be scaled up to decrease in-kind food donations. Cash assistance is used to support in-kind food provisions in emergency and camp settings in Greece (UNHCR 2016).

#### Promotion of breastfeeding

Breastfeeding encouragement and not automatically providing infant milk alternatives as food aid assistance is noted as a policy intervention in camp settings to improve child nutrition (UNHCR, IFE Core Group 2017). Breastfeeding is noted as a key recommendation to improve child nutrition in healthcare professional guidance for Greece (UNHCR 2016), Italy (UNICEF 2022) and the UK (Office for Health Improvement and Disparity 2014).

#### Other policies or interventions

Policies for the general improvement of nutrition in children, with migrant children noted as a priority group, include school canteen improvement measures in Italy (UNICEF 2022), Europe-wide physical activity strategies (implemented differently by country governments) (Schrier et al., 2019) and benefit support packages such as Healthy Start in the UK (Office for Health Improvement and Disparity 2014). Tackling obesity often remains an overarching policy priority for nutritional interventions (UNICEF 2022).

### Are policies implemented?

One document noted that food in hotels where asylum seekers are housed needs to meet nutrition standards set out by the Home Office for the UK (Agyemang and Van den Born, 2019). Another document noted that in Greece, a recent change of government meant that people (including minors) living in camps were denied food assistance and essential services due to issues relating to a lack of clarity on refugee status and lack of provisions (Smith, 2022).

Harkensee 2021 notes that, in the UK, challenges persist surrounding referral to nutrition services, with self-referral not an option, and often RAS families are not registered with general practitioners (GPs), meaning services may exist but go unused if local authorities do not refer people. Language barriers can also prevent effective implementation (Harkensee and Andrew, 2021).

### Impact of the interventions on the double burden of malnutrition

A UNHCR 2016 advisory paper is the only document to consider the impact of interventions on the double burden of malnutrition. It notes that people should receive food in line with their needs, including a limit on fat intake, noting that everyone should receive a food box tailored to their needs.

Other documents focused on undernutrition only.

### Recommendations to improve nutrition in the chosen population

Recommendations outlined in the documents studied include cash-based support for families (UNHCR 2018), citing increased independence and agency for families compared with providing ration provisions.

The data finds a recommendation around ensuring that people can access culturally appropriate food, which, in community settings, can include considering location and proximity to international food shops and, in provided accommodation, it's recommended that food is varied with some ingredients recognisable to people's country of origin (Bernharda et al., 2016).

There is a recommendation that healthcare professionals (HCPs) providing screening checks should ask about growth and development and perform a physical evaluation (Schrier et al., 2019).

Recommendations beyond the target population outline the need for effective nutrition and exercise strategies for all children delivered within a school environment (UNICEF 2022).

## Discussion

### Overview

The examined documents reveal limited policy diversity, often indirectly affecting the intended recipients due to interventions not tailored explicitly for RAS populations or children. Despite this, the documents tend to present broad recommendations for improving interventions.

While often recognising the significance of micronutrient requirements (UNHCR 2016; Bernharda et al., 2016; RCPCH 2018), the findings consistently reveal insufficiently tailored policies across the examined nations. Even when ostensibly comprehensive policies are implemented, conflicting reports suggest a misalignment between policy intent and practical outcomes.

Despite the acknowledged challenges and lack of a coherent picture, meaningful observations can be made:

### Missing puzzle pieces

The documents reviewed in this study appear to provide only a partial representation of the comprehensive nutrition landscape concerning RAS children. In practice, implementing policies within a given country is unlikely to align with their objectives consistently. While the intent of international guidelines may be acknowledged, practical application deviates from adherence. Conversely, it is plausible that localised initiatives and philanthropic aid are being deployed across various European regions to advance child nutrition within migrant communities. However, due to their informal nature, these initiatives remain largely undocumented, impeding the feasibility of comprehensive research and cross-comparisons.

### Challenges in deciphering policy

An identified limitation pertains to the complexity of discerning the legal status and nature of policy documentation solely through desk research. This challenge is particularly pronounced in international contexts, where search algorithms may introduce bias toward certain documents. This issue underscores broader concerns related to the opacity of policy formulation, highlighting the intricate dynamics of politics and legislative processes that make understanding and implementing policy so challenging.

This is further complicated by the political challenges of RAS communities being a population overlooked in law and policy, with a reliance on charity support for their health.

### Demand on services

The deficiency in interventions studied and the prevalence of inadequate nutrition among RAS children likely stem from a complex interplay of factors. These factors include excessive service demand, as evidenced by the substantial numbers of RAS, which frequently surpass initially projected capacity in many countries. This issue is particularly true in Greece (Asylum Information Database 2023). Moreover, this situation is exacerbated by the upward trajectory of living costs and the accompanying inflationary pressures on food prices.

Despite policies facilitating food access for RAS families, limited resources often impede prioritisation and achieving optimal nutrition in this group. Direct country-to-country comparisons of policies for RAS nutrition in children remain challenging due to the difference in need (number of eligible migrants), reporting (lack of knowledge about whether a policy is implemented) and lack of government resources. Limited journalistic reports of the situation in Greece and the UK have been found (Agyemang and Van den Born, 2019; Smith, 2022). Still, it is plausible that issues in other countries also exist but are less overt and more easily covered up; for example, RAS being housed in hotels rather than camps could make depictions of malnutrition less visible.

### NGO vs government policy

This research has found that in-country policies put in place by global agencies (namely UNHCR) through camps which external organisations run are more robust, more comprehensive and with more thought to complexities of nutrition and micronutrients than observed government-imposed policy. The only document in which data extraction brought information out for each of the four objectives set out by this research was a UNHCR policy document detailing policies in place in refugee camps run by UNHCR in Greece.

The role of NGOs in being non-political but with a policy influence (particularly in specific situations like emergencies) also speaks to the fragility of politics in emergencies and the ongoing need for NGOs.

### Food as politics

A general shift in the narrative about RAS in Europe over the past 20 years is observable, and it is plausible that some European nutrition policies act not to preserve, prolong and enrich life but to deter RAS from entering arrival countries. It is possible that some European governments could be directly or indirectly weakening nutritional interventions offered to RAS to discourage people from arriving. It may be that the political will to limit the numbers of RAS for multiple reasons is impeding the innovation, improvement and, ultimately, willingness to fund policies that would improve the lives and health of RAS.

### Breastfeeding recommendations are prominent

Recommendations for promoting high breastfeeding rates are consistently featured, particularly within global documents (UNICEF 2022; Prudhon et al., 2016; UNHCR 2016, Office for Health Improvement and Disparity 2014). It could be argued that this further demonstrates the limited political commitment to initiate, finance, and execute comprehensive nutritional policies. While breastfeeding offers undeniable public health advantages (World Health Organization 2018), particularly in resource-constrained environments where cleaning and safety pose challenges, it primarily places responsibility on mothers rather than authorities as a policy measure. It is a low-cost intervention that demands minimal planning, financial investment, or logistical arrangement, a convenience that further underscores the political reluctance to prioritise the subject.

### Lack of interventions on the double burden of malnutrition

Over-nutrition is not considered in the documents found in this study, meaning that the increasingly prevalent double burden of malnutrition over a life course is not being understood, considered, or addressed by policymakers. This is an example of where policy is not as advanced as the research – the double burden is well understood at all levels of the population (The Lancet 2019) and in RAS (Kumar et al., 2021), yet it is not mentioned in interventions.

### Lack of implementation data

The absence of implementation data presents challenges in evaluating the efficacy of existing interventions (Datta and Petticrew, 2013) and hinders impact assessment. For instance, documents suggest screening guidelines for HCPs dealing with RAS children upon arrival (Schrier et al., 2019, Office for Health Improvement and Disparity 2014, European Commission 2015, Bernharda et al., 2016, RCPCH 2018), yet there is an absence of standardised or objective metrics for assessing malnutrition.

The data shows that guidelines for migrant healthcare generally prioritise infectious disease monitoring and vaccination status (Office for Health Improvement and Disparity 2014, Schrier et al., 2019, Bernharda et al., 2016), meaning that nutrition screening and data capture often fall to HCP discretion and professional judgement. They are noting malnutrition ‘by eye’ rather than measurement. Whilst there is no direct evidence in this data that this widens the unmet need, it makes impact measurement and tackling life course ‘double burden of malnutrition’ challenging or impossible.

#### Intervention outcomes

Data on intervention outcomes, success and follow up over time is scarce, making meaningful assessment of the quality of intervention overtime challenging. As is often the case with policy, political change, change in staffing within settings rolling out the interventions, children getting older and moving away and not accessing services and lack of measurability of interventions by design are all factors which make outcomes hard to measure.

Confounding is also a challenge within nutrition measurement as there are several factors impacting individual nutrition over time.

#### Political, economic and social influence on policy

Policies responding to political and economic pressures often shape refugee nutrition strategies. Migration has become a highly charged issue in Europe, particularly with the rise of right-wing politics and global crises leading to increased numbers of displaced individuals seeking refuge. These factors have thrust migration and refugee concerns into the spotlight of European political discussions, underscoring the importance of addressing migrant nutrition policies. The lack of specific policies for migrant nutrition, especially for children and their families, highlighted in this review, underscores how RAS populations are often marginalised in political agendas. Despite being a focal point in political debates, RAS children lack tailored policy support, except through NGOs, leaving them disconnected from formal policy frameworks that cater to their unique needs.

### Limitations

Limitations of this study include lack of scope for translation of government articles and the UK focus of search engines used. For further research into interventions in this area, native speakers searching in the country where interventions took place would likely find richer results. Interviewing relevant country government representatives such as the Ministry of Health Public Health or nutrition leads would also provide a richer data set.

### Recommendations and conclusions

Table 4 below outlines a set of recommendations for a variety of sectors. The set of recommendations is designed to combine practicality, actionability, as well as be aspirational for key global stakeholders and national governments looking to tackle nutrition issues in RAS children outlined in this review.

**Table 4:** Recommendations for action based on the findings of this study split by governments, NGOs and further research.

**Recommendations**	
**For governments**	
**1**	**Consult NGOs on RAS nutrition policy**Global NGOs and United Nation's (UN) affiliated organisations such as the UN Refugee Agency have comprehensive and well adapted policies and documentation for refugee health, including nutrition, developed as part of their role in supporting emergencies in the past 50 years. Governments should enlist the support of these groups to utilise this experience and refer to these policies when developing domestic policies for RAS nutrition. This could be done in one or more of the following ways: • Reference global documents developed by NGOs for feeding in emergencies to ensure understanding of challenges and population need • Recruit an employee from the UN or other NGO to consult on RAS nutrition intervention planning • 3) Work directly with NGOs on the ground in the country policies are being developed for to ensure cohesion
**2**	**Use standardised measures when screening RAS children on arrival to destination countries** To enable impactful measurement and meaningful comparison, use World Health Organisation measures for nutrition, specifically mid-upper arm circumference (MUAC)-for-age to check for undernutrition (World Health Organization 2009). The introduction of standardised measures for defining malnutrition should accompany holistic, professional, judgement-based assessments to allow for sufficient records to be kept.
**3**	**Continue to invest in nutrition policies, including tackling obesity and providing mental health support for RAS throughout the life course, which would tackle the double burden of malnutrion, allowing for better long-term economic outcomes** Tackling poor nutrition in RAS children requires effective policies which acknowledge the life-course double burden of under and overnutrition. It's therefore important that nutrition policies are in place that impact people throughout their lives. This includes tackling obesity and providing mental health support for RAS, acknowledging that nutrition and mental health are often interlinked.
**4**	**Place RAS nutrition into the portfolio of health departments and ministries, separating it from immigration departments to minimise political bias impacting health policy** This ensures a separation between food and nutrition and politics around immigration ensuring that interventions are not weakened by politics and ensures that the Rights of the Child is upheld.
**5**	**Cross party consult on nutrition policies and mitigate against disruption in halting or amending policies due to political change** Minimise trends of RAS populations being politicised by actioning costed and relevant policies, advised by third parties which can sustainably be actioned for a generation, rather than political cycle.
**For NGOs / global agencies**	
**6**	***See recommendation 2 (use standardised measures when screening RAS children on arrival)***
**7**	**Develop quality standards for nutrition in RAS children and develop and roll out a league table from which to score countries in their improvements in managing nutrition for RAS** A quality standard developed by the World Health Organisation (WHO) and launched at a relevant policy summit such as United Nations General Assembly or Berlin World Health Summit which countries sign up and commit to would enable the WHO to call out countries which were falling behind against the outlined criteria and nudge political will toward improvement.
**8**	**Develop and streamline data management tools for nutrition, setting up a database of health measures for displaced people across Europe which could be accessed by country governments and healthcare providers** This would ensure that the double burden of malnutrition observable over a lifetime could be better managed and encourage governments to develop more comprehensive policies by seeing data and progress over time.
**For researchers**	
**9**	**Study the best ways to track, store and access data globally and in specific regions to allow governments to manage data collection and tracking more effectively** Extensive prevalence data exists around malnutrition, but it is currently disparate, making comparison for policymakers challenging. Acknowledging a better way of collection and storage of data, drawing from other clinical or policy areas would support governments and NGOs in this improvement.
**10**	**Continued study into the emergence of double and triple burden of malnutrition in RAS populations** This will illuminate the problem and ensure that governments develop interventions with a longer-term perspective informed by the life course approach. In this emerging area of nutrition study, more data on double and triple burden in RAS is still needed.

## Conclusion

This study underscores well-documented nutritional concerns among migrant children, particularly recent settlers, and those with undocumented or asylum-seeking status, in Europe. The European context serves as an insightful case study for refugee and asylum food policy due to the escalating numbers of migration and divergent political approaches across nations, enabling fruitful comparisons. Unlike countries such as Australia, Canada, and the US, which predominantly attract migrants from more limited source countries (Connor et al., 2019), Europe witnesses global migration routes, resulting in distinctive health and nutrition challenges. Despite Europe providing a fascinating spotlight, observations of this study hold relevance for all countries hosting RAS children and families.

The principal discovery of this study is the lack of transparency regarding the extent and specifics of policies in place, leading to unaddressed nutritional need among RAS children across Europe. This lack of clarity is likely rooted in the challenging political landscape and controversies surrounding the scale, placement, and economic implications of RAS populations in Europe. Despite an emerging prevalence of the lifetime double burden of malnutrition within this demographic (Ankomah et al., 2022), there is an absence of interventions aimed at addressing this issue. As nations navigate strategies to manage obesity, which is more politically prominent than nutritional policies for RAS populations, strategies for identifying and mitigating this dual burden should be considered.

Advocating for strategies that address the dual burden of malnutrition could present a politically acceptable avenue for effecting change in the realm of nutrition for RAS children. By emphasising the complex interplay between undernutrition and overnutrition, policymakers may find an approach that resonates across various political perspectives and garners broader support. This approach recognises that RAS children often face a unique amalgamation of nutritional challenges, necessitating a comprehensive policy response that transcends conventional classifications of malnutrition. Therefore, exploring strategies encompassing both aspects of the dual burden might foster a more inclusive and cooperative discourse among stakeholders, leading to more effective policy formulation and implementation and thus improving nutrition outcomes. Overall, establishing effective policies to address RAS nutrition challenges and confronting the life course double-burden of malnutrition in this group ideally necessitates the separation of nutritional considerations from political ones. Where politics and nutrition remain entwined, focusing interventions on addressing the double burden could be a practical policy option.

## CRediT authorship contribution statement

**Rebecca Lawes:** Writing – original draft, Conceptualization, Data curation, Formal analysis, Investigation, Methodology, Project administration. **Professor Debbi Marais:** Supervision, Validation, Writing – review & editing. **Professor Mariza Louw:** Supervision, Writing – review & editing, Project administration. **Ms Beatrice Bennett:** Data curation, Formal analysis.

## Declaration of competing interest

The authors declare that they have no known competing financial interests or personal relationships that could have appeared to influence the work reported in this paper.

## References

[bib0028] Adams J. (2016). Searching and synthesising ‘grey literature’ and ‘grey information’ in public health: critical reflections on three case studies. Syst Rev.

[bib0020] Agyemang C, Van den Born BJ. (2019). Non-communicable diseases in migrants: an expert review. J. Travel Med..

[bib0003] Ankomah A (2022). Double burden of malnutrition among migrants and refugees in developed countries: a mixed-methods systematic review. PLoS One.

[bib0027] Arksey H, O'Malley L (2005). Scoping studies: towards a methodological framework. Int. J. Soc. Res. Methodol..

[bib0043] Asylum Information Database. 2023. Overview of the main changes since the previous report update. Available from: https://asylumineurope.org/reports/country/greece/overview-main-changes-previous-report-update/. Last accessed 21 August 2023.

[bib0019] Berggreen-Clausen A. (2022). Food environment interactions after migration: a scoping review on low- and middle-income country immigrants in high-income countries. Public Health Nutrition.

[bib0035] Bernharda S. (2016). Guidance for testing and preventing infections and updating immunisations in asymptomatic refugee children and adolescents in Switzerland. Paedeatrica.

[bib0009] Concern Worldwide. 2023. The 10 largest refugee crises to know in 2023. Available from: https://www.concern.net/news/largest-refugee-crises. Last accessed 14 August 2023.

[bib0049] Connor, P. et al. 2019. How European and U.S. unauthorized immigrant populations compare. Available from: https://www.pewresearch.org/short-reads/2019/11/13/how-european-and-u-s-unauthorized-immigrant-populations-compare/#:∼:text=Unauthorized%20immigrants%20living%20in%20Europe,Europe's%20proximity%20to%20many%20regions. Last accessed 14 August 2023.

[bib0046] Datta J., Petticrew M. (2013). Challenges to evaluating complex interventions: a content analysis of published papers. BMC Public Health.

[bib0004] Di Daniele N. (2019). The role of preventive nutrition in chronic non-communicable diseases. Nutrients.

[bib0047] European Commission. 2015. Handbook for health professionals: health assessment of refugees and migrants in the EU/EEA. Available from: https://health.ec.europa.eu/system/files/2016-11/handbook_healthprofessionals_en_0.pdf. Accessed 21 August 2023.

[bib0012] Eurostat. 2023. Children in migration - asylum applicants. Available from: https://ec.europa.eu/eurostat/statistics-explained/index.php?title=Children_in_migration_-_asylum_applicants#Main_features_at_EU_level_in_2022 Accessed 21 August 2023.

[bib0022] Fabio M. (2014). Nutrition for refugee children: risks, screening, and treatment. Current Problems in Paediatric and Adolescent Health Care.

[bib0024] Fazel M, Stein A. (2002). The mental health of refugee children. Arch. Dis. Child..

[bib0018] Gilbert P.A., Khokhar S. (2008). Changing dietary habits of ethnic groups in Europe and implications for health. Nutr. Rev..

[bib0015] Gingell T. (2022). Determinants of food security among people from refugee backgrounds resettled in high-income countries: a systematic review and thematic synthesis. PLoS One.

[bib0017] Gualdi-Russo E. (2014). Obesity and physical activity in children of immigrants. Eur. J. Public Health..

[bib0042] Harkensee C, Andrew R. (2021). Health needs of accompanied refugee and asylum-seeking children in a UK specialist clinic. Acta Paediatr..

[bib0001] Royal College of Paediatrics and Child Health. 2018. Refugee and asylum seeking children and young people - guidance for paediatricians. Available from: https://www.rcpch.ac.uk/sites/default/files/generated-pdf/document/Refugee-and-asylum-seeking-children-and-young-people—guidance-for-paediatricians.pdf. Accessed 21 August 2023.

[bib0030] Intersos GR. 2023. Being Hungry in Europe: an analysis of the food insecurity experienced. Available from: https://www.intersos.gr/wp-content/uploads/2023/05/Report-_Being-Hungry-in-Europe_3.pdf. Last accessed 21 August 2023.

[bib0025] ISSOP Migration Working Group (2018). ISSOP position statement on migrant child health. Child Care Health Dev..

[bib0023] Kadir Ay (2019). Children on the move in Europe: a narrative review of the evidence on the health risks, health needs and health policy for asylum seeking, refugee and undocumented children. *BMJ paediatrics* open.

[bib0016] Krishnamani P. (2016). Undernutrition in Refugee Children. Delaware J. Public Health.

[bib0007] Kumar P. (2021). Prevalence and factors associated with triple burden of malnutrition among mother-child pairs in India: a study based on National Family Health Survey 2015–16. BMC Public Health.

[bib0021] Lopriore C. (2004). Spread fortified with vitamins and minerals induces catch-up growth and eradicates severe anaemia in stunted refugee children aged 3–6 y. Am. J. Clin. Nutrit..

[bib0008] Nisbet C (2022). Food Security Interventions among Refugees around the Globe: a Scoping Review. Nutrients.

[bib0005] Nyaradi A. (2013). The role of nutrition in children's neurocognitive development, from pregnancy through childhood. Front Hum Neurosci.

[bib0033] Office for Health Improvement and Disparity. 2014. UK Government guidance - Children's health: migrant health guide. Available from: https://www.gov.uk/guidance/childrens-health-migrant-health-guide. Accessed 21 August 2023.

[bib0044] Prudhon C. (2016). Research priorities for improving infant and young child feeding in humanitarian emergencies. BMC Nutr.

[bib0036] RCPCH: Refugee and asylum-seeking children and young people - guidance for paediatricians. 2018. https://www.rcpch.ac.uk/sites/default/files/generated-pdf/document/Refugee-and-asylum-seeking-children-and-young-people—guidance-for-paediatricians.pdf.

[bib0034] Schrier L. (2019). Medical care for migrant children in Europe: a practical recommendation for first and follow-up appointments. Eur. J. Pediatr..

[bib0041] Smith, Helen. 2022. Greek government blamed for hunger crisis in refugee camps. Available from: https://www.theguardian.com/world/2022/jan/24/greek-government-blamed-for-hunger-crisis-in-refugee-camps#:~:text=Humanitarian%20groups%20have%20accused%20the,thousands%20unable%20to%20access%20food. Accessed 21 August 2023.

[bib0029] Taylor Diane. (2023). Children in England's asylum hotels suffering from malnutrition. Guardian.

[bib0014] Terragni L. (2014). Migration as a turning point in food habits: the early phase of dietary acculturation among women from south asian, african, and middle eastern countries living in norway. Ecol. Food Nutr..

[bib0006] The Lancet, 2019. The Double burden of malnutrition. Available from: https://www.thelancet.com/series/double-burden-malnutrition. Accessed 21 August 2023.

[bib0026] Tricco AC (2018). PRISMA extension for scoping reviews (PRISMA-ScR): checklist and explanation. Ann. Intern. Med..

[bib0032] UNHCR. 2016. Food and Nutrition Operational Considerations for Refugee and Migrant settings in Greece. Available from: https://data.unhcr.org/en/documents/details/47797. Last accessed 21 August 2023.

[bib0037] UNHCR. 2016. Food and Nutrition Operational Considerations for Refugee and Migrant settings in Greece. Available from: https://data.unhcr.org/en/documents/details/47797. Last accessed 21 August 2023.

[bib0039] UNHCR. 2016. Food and Nutrition Operational Considerations for Refugee and Migrant settings in Greece. Available from: https://data.unhcr.org/en/documents/details/47797. Last accessed 21 August 2023.

[bib0031] UNHCR. 2018. Multi-Purpose Cash and Sectoral Outcomes - Case Study Greece. Available from: https://www.unhcr.org/media/multi-purpose-cash-and-sectoral-outcomes-case-study-greece. Last accessed 21 August 2023.

[bib0038] UNHCR, IFE Core Group. 2017. Infant and Young Child Feeding in Emergencies – Operational Guidance on Infant Feeding in Emergencies. Available from: https://www.ennonline.net/attachments/3127/Ops-G_English_04Mar2019_WEB.pdf. Last accessed 21 August 2023.

[bib0040] UNICEF. 2022. Deep dive - Child Guarantee - Analysis of politics, programmes, budgets addressing child poverty and social exclusion in Italy. Available from: https://www.unicef.org/eca/media/23011/file/Deep%20Dive%20brief%20en.pdf. Last accessed 21 August 2023.

[bib0002] UNICEF. 2023. Emergencies Programme. Refugee and migrant children in Europe. Available from: https://www.unicef.org/eca/emergencies/refugee-and-migrant-children-europe. Last accessed 14 August 2023.

[bib0011] United Nations High Commission for Refugees. 2019. UNHCR projected global resettlement needs 2020. Available from: https://www.unhcr.org/en-au/protection/resettlement/5d1384047/projected-global-resettlement-needs-2020.html. Last accessed 21 August 2023.

[bib0010] United Nations High Commissioner for Refugees. 2023. Climate change and disaster displacement. Available from: https://www.unhcr.org/media/climate-change-and-disaster-displacement-global-compact-refugees. Last accessed 21 August 2023.

[bib0013] Wood J. (2021). What factors are associated with food security among recently arrived refugees resettling in high-income countries? A scoping review. Public Health Nutr..

[bib0048] World Health Organization. 2009. WHO child growth standards and the identification of severe acute malnutrition in infants and children. Available from: https://apps.who.int/iris/bitstream/handle/10665/44129/9789241598163_eng.pdf Last accessed 14 August 2023. 24809116

[bib0045] World Health Organization. 2018. Breastfeeding. Available from: https://www.who.int/news-room/facts-in-pictures/detail/breastfeeding. Last accessed 16 August 2023.

